# The Anchorage of Bone Cells onto an Yttria-Stabilized Zirconia Surface with Mild Nano-Micro Curved Profiles

**DOI:** 10.3390/dj8040127

**Published:** 2020-11-10

**Authors:** Susanne Staehlke, Armin Springer, Thomas Freitag, Jakob Brief, J. Barbara Nebe

**Affiliations:** 1 Department of Cell Biology, University Medical Center Rostock, D-18057 Rostock, Germany; susanne.staehlke@med.uni-rostock.de (S.S.); thomas.freitag@uni-rostock.de (T.F.); 2 Medical Biology and Electron Microscopic Center, University Medical Center Rostock, D-18057 Rostock, Germany; Armin.Springer@med.uni-rostock.de; 3 VITA Zahnfabrik H. Rauter GmbH & Co. KG, D-79713 Bad Säckingen, Germany; J.Brief@vitaclinical.com

**Keywords:** dental ceramic implant, zirconia, nano-micro topography, EDX, scanning electron microscopy, osteoblasts, cell anchorage, in vitro, ROS, ATP

## Abstract

The high biocompatibility, good mechanical properties, and perfect esthetics of ceramic dental materials motivate investigation into their suitability as an endosseous implant. Osseointegration at the interface between bone and implant surface, which is a criterion for dental implant success, is dependent on surface chemistry and topography. We found out earlier that osteoblasts on sharp-edged micro-topographies revealed an impaired cell phenotype and function and the cells attempted to phagocytize these spiky elevations in vitro. Therefore, micro-structured implants used in dental surgery should avoid any spiky topography on their surface. The sandblasted, acid-etched, and heat-treated yttria-stabilized zirconia (cer.face^®^14) surface was characterized by scanning electron microscopy and energy dispersive X-ray. In vitro studies with human MG-63 osteoblasts focused on cell attachment and intracellular stress level. The cer.face 14 surface featured a landscape with nano-micro hills that was most sinusoidal-shaped. The mildly curved profile proved to be a suitable material for cell anchorage. MG-63 cells on cer.face 14 showed a very low reactive oxygen species (ROS) generation similar to that on the extracellular matrix protein collagen I (Col). Intracellular adenosine triphosphate (ATP) levels were comparable to Col. Ceramic cer.face 14, with its sinusoidal-shaped surface structure, facilitates cell anchorage and prevents cell stress.

## 1. Introduction

In Germany, more than 1.3 million artificial teeth are implanted annually [1]. Although titanium (Ti) and its alloys are still the most-used dental implant materials, there are a number of reasons they might be progressively replaced by ceramic implants in the future. Despite being very biocompatible relative to other metals such as Co, Ni, and Fe [2], Ti is released in the presence of biological fluids and tissues [3,4]. An increased number of case reports described adverse reactions to Ti-based alloys [3], and 10–15% of patients expressed genetically disposed titanium oxide incompatibility. This is important because Ti particles surrounding peri-implant tissues are a common finding [4]. The particles were mostly around the implants and inside epithelial cells, connective tissue, macrophages, and bone. The causes of Ti release could be friction during implant insertion or corrosion of the implant surface i.a. [4]. A patient survey in 2019 with 1024 patients revealed that 74% of patients chose ceramic implants [5]. Ceramic implants provide an esthetic benefit for patients [6], in contrast to Ti, where discoloration of the peri-implant soft tissue may occur [7]. After wearing the implants for years it could be possible that the gingiva reduced their level and parts of the implant could be visible or shining through. In a clinical study by Cosgarea et al. [8], multi-spectral images of the peri-implant soft tissues and the gingiva of the neighboring teeth were taken with a colorimeter. The authors revealed that the peri-implant soft tissue around zirconia demonstrated a better color match to the soft tissue at natural teeth than titanium. The development of ceramic materials for dental implants has been accelerated in recent years, and dental implants made of zirconia have been commercially available for several years [9]. The share of the market will increase from nearly 1% in 2016 up to 9% in 2025 [10]. To date, it can be stated that zirconia is a versatile material for implant prosthodontic application, but longer-term multi-centered studies are needed to assess success criteria [6]. Especially, the initial phase of a dental implant in the oral environment seems to be critical since implant loss was mostly reported within the first year, especially within the healing period. Thereafter, nearly constant survival curves were observed [9]. The requirement for the clinical success of a dental implant is the permanent osseointegration, i.e., the formation of a direct bone-implant contact along the endosseous part [11]. The major challenge for materials science is to provide biomaterials that enable optimum performance in the human organism. The occupation of a biomaterial surface by the osteoblasts in vitro is divided into stages, beginning with the initial adhesion of the cells, their spreading, and their migration along the topography, followed by proliferation and cell differentiation [12]. Besides chemical gradients [13,14,15,16], the topography is a decisive factor for cell guidance and anchorage. Whereas micro-indentations let the cells aggregate in the cavities (Ø 100 µm) [17] or direct the cell migration into the concave region via curvotaxis [18], fabricated nano-micro grooves pushed the cells’ alignment [19,20]. Furthermore, square pyramids elongated the cell form and directed the growth because cells could not find optimal contact areas due to the peak geometry [21]. We revealed earlier that topographies with sharp edges changed not only the overall cell morphology and intracellular actin cytoskeleton formation [22] but also hampered diverse cell functions. Edges, ridges, crevices, and points on titanium surfaces provoked an increase in reactive oxygen species (ROS) in cells [23], reduced the intracellular adenosine triphosphate (ATP)-content [24], and decelerated calcium ion mobilization from intracellular stores [25]. In addition, the wnt-signaling pathway was disturbed, i.e., we observed increased expression of inhibitors like ICAT (inhibitor of β-catenin and transcription factor-4) [23]. Not least, the cells attempted, unavailingly, to phagocytize these elevations, which led to cellular stress followed by impaired synthesis of extracellular matrix proteins [24]. These in vitro findings were irrespective of the mode of the surface design, which was stochastically or geometrically produced. There are many ways to topographically modify ceramic surfaces such as polishing, airborne particle abrasion with alumina (sandblasting), acid etching with hydrofluoric acid or sulfuric acid followed by heating, as well as selective infiltration etching (i.e., the coating of the material surface with a specific infiltration glass and heating [26]). Ceramic (zirconia) can also be treated with laser or ultraviolet light; the latter induces a superhydrophilic surface [26]. From our observations, we concluded that micro-structured material implants used in dental surgery should avoid any spiky topographical heights on their surface. A recently developed ceramic dental implant material revealed a surface free of spikes and sharp edges. Sandblasted, acid-etched, and heat-treated yttria-stabilized zirconia (cer.face^®^14) was cell stimulating and displayed a strong enhancement of the osteocalcin mRNA level [27]. Osteocalcin is a peptide synthesized in odontoblasts of the teeth and in osteoblasts of the bones with an affinity to hydroxyapatite and calcium. It is a major non-collagenous protein generally involved in bone matrix organization and occurs late in the mineralization process [28].

The objectives of this in vitro study were to evaluate if the ceramic cer.face 14 surface allows bone cells stress-free attachment, growth, and anchorage with the nano-micro curved surface profile.

## 2. Materials and Methods

### 2.1. Materials: Yttria-Stabilized Zirconia

Disks of yttria-stabilized zirconia with a diameter of 13 mm and a thickness of 1.5 mm were micro-structured by sandblasting (P-G 400, Harnisch and Rieth, Winterbach, Germany) with 105 µm alumina particles (Hasenfratz no. 120, Assling, Deutschland) at a pressure of 6 bars. The specimens were etched for 1 h in 40% hydrofluoric acid (HF), thoroughly rinsed in distilled water for 5 min, and finally heat treated at 1250 °C (LH 15/14, Nabertherm, Lilienthal, Germany) [11,27]. The resulting surface is named **cer.face^®^14** by the company (vitaclinical, Vita Zahnfabrik, Bad Säckingen) and is already being used in dental implant surgery. The water contact angle (WCA) of cer.face 14 was determined earlier using the sessile drop technique by a drop shape analyzer (DSA100) with water and diiodomethane [11]. The roughness was measured by the contact profilometer (T1000/TKK50). The cer.face^®^14 surface features a wettability of 96° (water contact angle) and a roughness of 1.16 µm (Ra, arithmetical mean height) [11].

*EDX analysis*: elemental composition of the tested ceramic dental material (cer.face^®^14) was done by EDX spectroscopy (detector: XFlash 6/30 of the FE-SEM MERLIN^®^ VP Compact, Co. Zeiss, Oberkochen, Germany). Quantification analysis (software: Quantax400, Co. Bruker, Berlin, Germany) was carried out on the basis of the measured spectra of a specific area; it is given as percentage of mass (standardized).

### 2.2. Cell Culture

*Cells*: One of the most popular cell lines in osteogenesis studies is the human osteoblastic line MG-63. Cell physiology processes during the first 24 h, including cell morphology, availability of adhesion receptors, cell cycle phases, and the expression of signaling proteins, remained stable over the entire range of passages 5–30 [29,30]. Therefore, experiments were performed with human MG-63 osteoblasts (ATCC^®^ CRL-1427™; American Type Culture Collection, Manassas, VA, USA), which were cultured in Dulbecco’s modified Eagle’s medium (DMEM) (Life Technologies GmbH, Darmstadt, Germany) containing 10% fetal calf serum (FCS) (Biochrom FBS Superior, Merck KgaG, Darmstadt, Germany; EU-approved) and 1% gentamicin (Ratiopharm GmbH, Ulm, Germany) at 37 °C in a humidified atmosphere with 5% CO_2_.

*Collagen coating*: Collagen type I is the most abundant extracellular matrix protein in mammals and provides a biocompatible environment for cells. Therefore, it is used as a matrix for tissue engineering or as implantable medical product [31]. Because collagen I serves as biological matrix with best preconditions for stress-free cell attachment the control experiments for adenosine triphosphate (ATP), reactive oxygen species (ROS) and DAPI staining (see Section 2.3) were done on collagen I (**Col**)-coated glass substrates as described by Mörke et al. [19]. In brief, collagen I (rat tail tendon, 20 mg/cm^2^, BD Biosciences, Heidelberg, Germany) was diluted to 200 µg/mL in 0.1% acetic acid (Sigma-Aldrich, Munich, Germany). The Col solution (0.1 mg/mm^2^) was dropped on the glass samples (Ø 12 mm, Menzel GmbH, Braunschweig, Germany) and allowed to dry overnight under sterile conditions in a laminar flow box. Before use, samples were rinsed three times with phosphate buffer solution (PBS, Sigma-Aldrich).

### 2.3. Stress Level of Cells

*ATP*: MG-63 osteoblasts (5.2 × 10^4^) were cultured for 24 h on the samples. Intracellular free ATP was determined using the ATP assay kit (Abcam, Cambridge, MA, USA) following the manufacturer’s instructions for fluorometric assays. For the cell lysate preparation, cells were harvested from the samples by 0.05% Trypsin-EDTA (ethylenediaminetetraacetic acid; PAA Laboratories, Pasching, Australia), washed with cold PBS, and resuspended in assay buffer. After final centrifugation (13,000× *g*, 4 °C, 5 min), the supernatant was collected. ATP reaction mix was added to each standard- (duplicate) and sample-well (triplicate) in a black 96-well plate (Greiner Bio-One International GmbH, Kremsmünster, Austria). After 30 min incubation, the fluorescence was measured by the microplate reader (Tecan infinite M200; Tecan i-control, 1.9.17.0) [24]. The concentration of intracellular ATP was determined for cer.face14, compared to Col controls, in three independent experiments.

*DAPI*: After the trypsination process to obtain the cells for ATP analysis, the sample surfaces were examined with regard to still-anchored cell residues. Surfaces were fixed with 4% paraformaldehyde (PFA; Sigma-Aldrich) for 10 min at room temperature, washed with PBS, and mounted using Fluoroshield™ with DAPI (40,6-diamidino-2-phenylindole; 1.5 g/mL, Sigma-Aldrich). The strength of cell adhesion after trypsination on cer.face14 surfaces compared with Col controls was determined in three independent experiments (à 5 images via confocal scanning microscopy, LSM 780, Carl Zeiss). The number of cell nuclei per image was determined using ImageJ.

*ROS*: Intracellular ROS generation was evaluated using 20,7′-dichlorofluorescein diacetate (DCF-DA cellular ROS detection assay kit, Abcam). For this purpose, MG-63 cells were trypsinated and the pellet was incubated in the DCF-DA dye solution (20 µM) at 37 °C for 30 min in the dark. Stained MG-63 cells (5.2 × 10^4^) were then cultured in complete DMEM (without phenol red; Life technologies GmbH) for 1 and 24 h on the samples. The fluorescence intensity of DCF, oxidized in the presence of ROS, was determined by the microplate reader (Tecan infinite 200). The cer.face 14 ceramic was compared with a Col-coated glass substrate (3 independent experiments). As positive control H_2_O_2_ treated MG-63 cells was used (50 mM tert-butyl hydrogen peroxide, 1 h before staining), which shows the maximum increase in these DCF-DA stained osteoblasts.

### 2.4. Image Analysis

*Scanning electron microscopy*: Material surfaces and samples with cells (á 2 independent approaches) were analyzed by a field emission scanning electron microscope (FE-SEM, MERLIN^®^ VP Compact) equipped with an energy dispersive X-ray (EDX) detector (XFlash 6/30) and analysis software (Quantax400, Co. Bruker, Berlin, Germany).

MG-63 cells were washed with PBS, fixed with 2.5% glutardialdehyde (Merck) for 1 h at RT, and stored at 4 °C overnight. Fixed samples were washed with Na-phosphate buffer (0.1 M), dehydrated in an ascending series of ethanol, and critical point dried (Emitech K850, Co. Quorum Technologies LTD, East Sussex, UK). Samples were mounted on Aluminum SEM-carrier with adhesive conductive carbon tape (PLANO, Wetzlar) and coated with carbon under a vacuum (EM SCD 500, Co. Leica, Bensheim, Germany).

In order to analyze the contact sides of MG-63 cells with the material surface, the dried samples were broken with two grippers and mounted on Aluminum SEM-carrier with adhesive conductive carbon tape at an angle of about 75° and then carbon coated.

*Confocal laser scanning microscopy*: Fluorescence images were taken from an inverted confocal laser scanning microscope LSM 780 (Carl Zeiss, Jena, Germany) equipped with a diode laser (405 nm). The ZEISS objective (Plan-Apochromat 10×, 0.3 M27/AA 2.0 mm/0.17 mm) and the ZEN 2011 (black version) software (Carl Zeiss) were used for acquisition. To determine the number of nuclei, DAPI-stained cells (ex359/em457 nm) were displayed on the samples at maximal pinhole. Representative images were taken from at least three independent experiments.

### 2.5. Statistics

Statistical analyses were conducted with the GraphPad Prism7 software (GraphPad Software Inc., La Jolla, CA, USA). Results represented as graphs were expressed as mean ± s.e.m. (standard error of the mean), and DAPI values were expressed as mean ± standard deviation. Data analysis was conducted using paired tests: one-way ANOVA posthoc uncorrected Fishers LSD (for ROS) and Wilcoxon matched-pairs signed rank test (for ATP and DAPI). *p*-values < 0.05 were recorded as an indication of significant differences.

## 3. Results

### 3.1. Surface Characteristics

**Figure 1:**
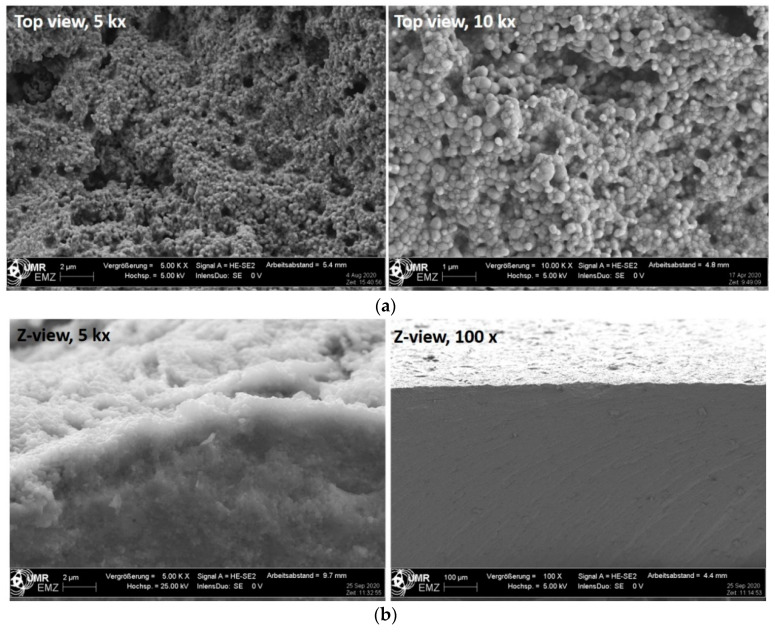
Scanning electron microscopy of the cer.face 14 ceramic. (**a**) Surface topography. Note a landscape with nano-micro hills and cavities, nearly sinusoidal-shaped, (bars: left 2 µm, right 1 µm). (**b**) Cross section of the material. No different layers were apparent, (bars: left 2 µm, right 100 µm). (FE-SEM Merlin, Carl Zeiss).

**Figure 2:**
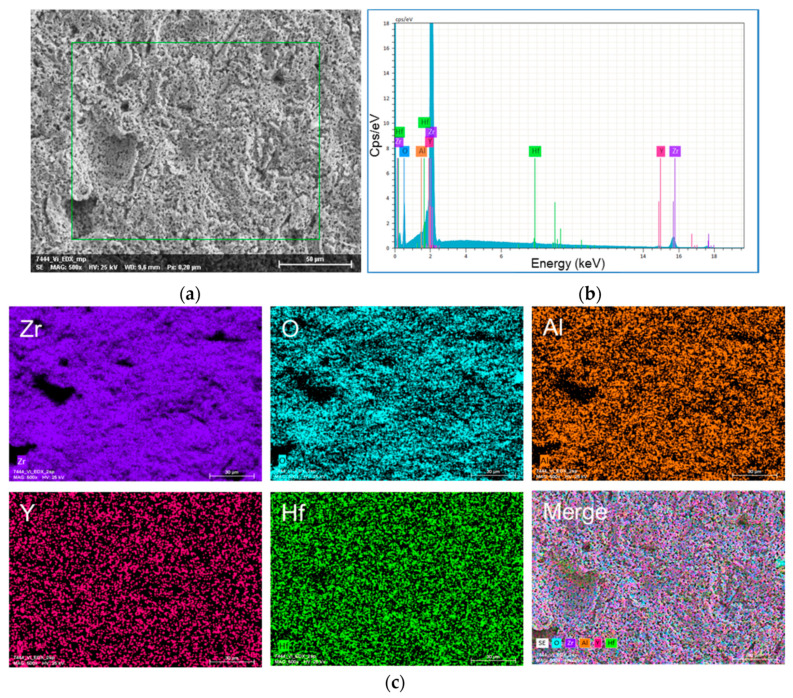
EDX analysis of the cer.face 14 surface. (**a**) Topographical scan, bar 50 µm, 500×; (**b**) Elemental composition; (**c**) In-depth analysis, bars 30 µm, main components Zr, O, Y, Hf, and Al.

**Figure 3:**
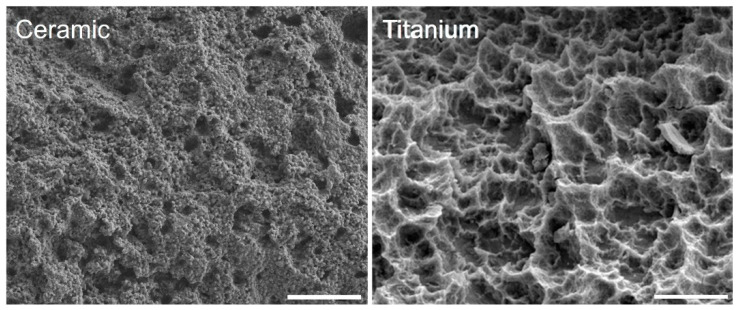
Scanning electron microscopy of dental materials: comparison of ceramic (Material: Vita, zirconia, acid HF; SEM: FE-SEM Merlin, Carl Zeiss, bar 7 µm, 3.000×) versus titanium (Material: Straumann, Ti grade 4, acid HCL and H_2_SO_4_; SEM: DSM 960A, Carl Zeiss, 10 kV, 3.3 A, bar 7 µm; adopted from Duske K et al. [32]) both sandblasted and acid-etched. Note that the resulting topographical features seem to depend on the basic materials.

The surface topography of the yttria-stabilized zirconia cer.face 14 is shown in Figure 1. After sandblasting, acid etching, and the final heat treatment of the zirconia surface, sinusoidal-shaped, nano-micro structured features can be observed via FE-SEM. The cross section via FE-SEM shows the nano-topography on top and did not reveal separated material layers. The in-depth analysis using the EDX detector of the FE-SEM revealed the following main components of this ceramic surface (in % of mass): Zr 72.77, O 22.37, Y 3.24, Hf 1.43, and Al 0.19, (total: 100) (Figure 2). Interestingly, this elemental composition was also found in the cross section (lateral direction) (Figure 1b, right). Sandblasting and acid etching (via HF) of the zirconia surface resulted in a semi 3D nano-micro topography. Comparing ceramic and titanium landscapes after the application of nearly the same surface treatment processes (for Ti HCL and H_2_SO_4_), a different topographical landscape can be observed (Figure 3).

### 3.2. Cell Morphology

**Figure 4:**
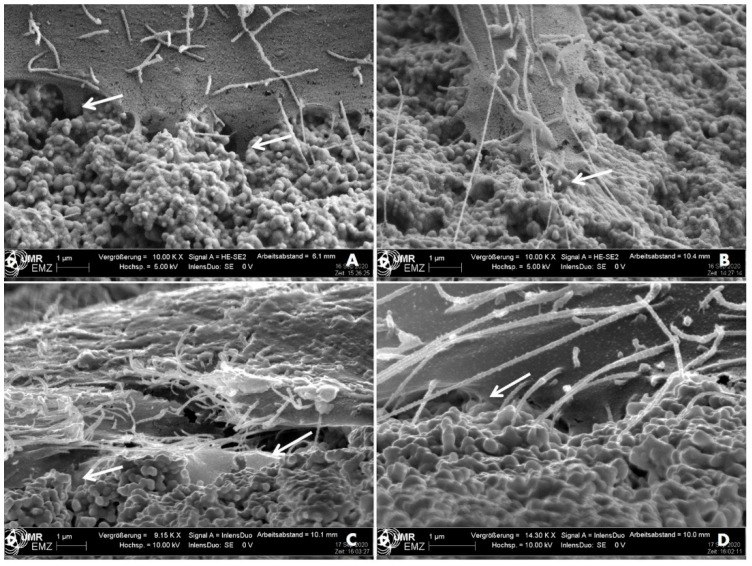
Bone cell anchorage onto the cer.face 14 ceramic. Note that cells are strongly interconnected with the nano-micro curved, sinusoidal-shaped profiles of the ceramic surface after 24 h (arrows). Detection via scanning electron microscopy (FE-SEM Merlin, Carl Zeiss, A + B angel 21°, HE-SE detector, C + D angel 75°, InlenseDuo detector; scale bars 1 µm, magnification (**A**–**C**) ~10 kx, (**D**) ~14 kx).

One aspect of cell-material interaction is the topographically induced cell behavior. In this paper, the focus was solely on the anchorage of cells to the nano-micro curved topography. The electron microscopic studies after 24 h revealed that MG-63 cells were intensely interconnected with the nano-micro profiles of the cer.face 14 ceramic (Figure 4). The MG-63 osteoblasts were able to anchor themselves with their filopodia, which are thin membrane protrusions which probe the surroundings. The diameters of the cells’ filopodia as well as the substructure of the ceramic surface are both in the nanometer range. Therefore, in the transition zone between the cell margin and the ceramic nano-hills, both structures are hardly to be distinguished from each other (Figure 4B).

**Figure 5:**
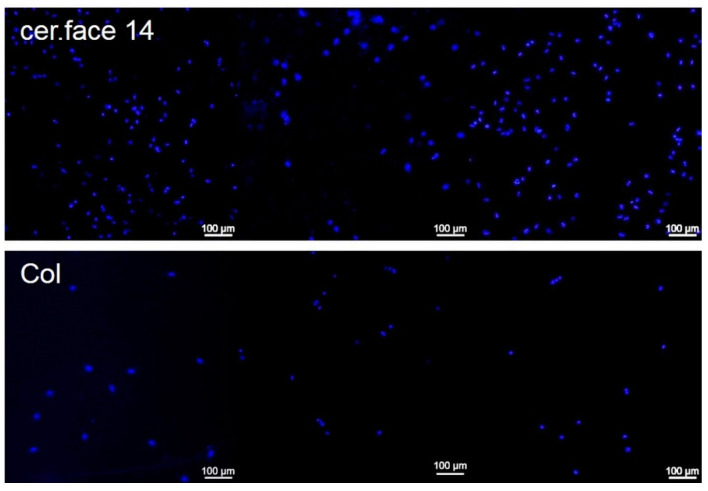
Cell adhesion on the cer.face 14 ceramic. Note that a lot of residual, adhered cells (nuclei in blue, DAPI staining) can be seen that withstood the normal trypsination process due to their embedding in the nano-micro structured surface (n = 180 ± 90); in comparison, cells on a Col-coated plane surface are removed nearly completely (n = 26 ± 14), which is significant with *p* < 0.01. Detection via confocal scanning microscopy (LSM 780, Carl Zeiss, sale bars 100 µm).

The DAPI-staining of cell nuclei clearly showed that the MG-63 osteoblasts were tightly attached to the nano-micro profile of the cer.face 14 surface. The typical detachment of the cells by trypsin was insufficient to remove the cells completely, as is the case with a Col-coated, plane surface (Figure 5), 180 ± 90 cells vs. 26 ± 14 cells (*p* < 0.01), respectively. On the Col control, cells were not so strongly attached as on cer.face 14, although the cells’ adhesion receptors could find ligands of the extracellular matrix component collagen I.

### 3.3. Stress Level of Cells

**Figure 6:**
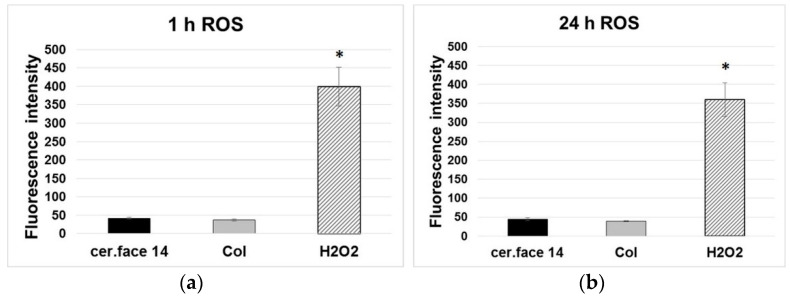
Reactive oxygen species (ROS): non/negligible stress level of osteoblasts on ceramic (cer.face 14), which is comparable with cells on the extracellular matrix protein collagen I (Col) (**a**) after 1 h, (**b**) after 24 h. Cells’ intracellular ROS level is ~9 times higher if stressed with H_2_O_2_ as positive control. The amount of ROS in human osteoblasts MG-63 was detected via DCF-DA cellular ROS detection assay kit (Tecan Infinity 200). Statistics: one-way ANOVA posthoc uncorrected Fishers LSD, significance * *p* < 0.05, n = 3 (independent experimental approaches).

In this study, mild nano-micro curved profiles, which led to an improved anchorage of the MG-63 cells to the surface, did not cause intracellular stress. To investigate the ROS production within the first 24 h, the DCF-DA ROS detection assay was used. It indicated negligible ROS levels in MG-63 cells on cer.face 14, comparable with the Col control (~0.9-fold) (Figure 6). In contrast, H_2_O_2_ as internal positive control and inducer of intracellular ROS led to a strong and significant increase of the DCF fluorescence at 1 and 24 h (~9-fold). An increased generation of ROS as an early marker for cellular stress was not detectable in the MG-63 osteoblasts on cer.face 14 with the mild nano-micro curved profiles within 24 h.

**Figure 7:**
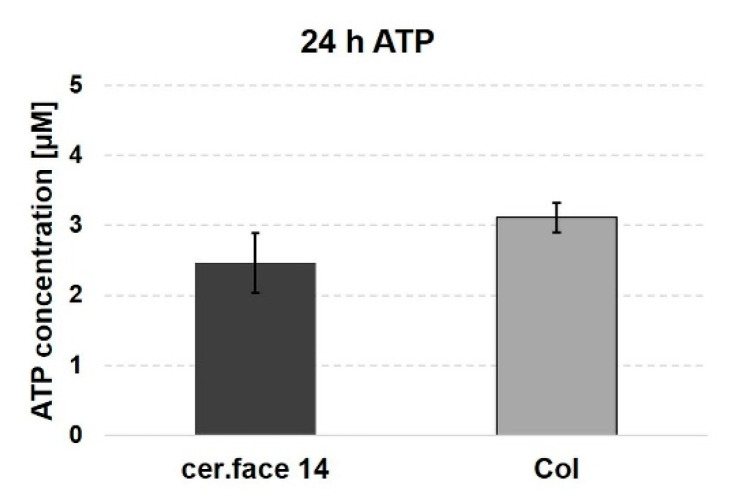
Adenosine triphosphate (ATP): the amount of energy-rich molecules in osteoblasts on ceramic (cer.face 14) is comparable with cells on collagen-coated surfaces (Col). Intracellular free ATP was detected via fluorometric ATP assay kit (Tecan Infinity 200). Statistics: Wilcoxon matched-pairs signed rank test, significance n.s. at *p* < 0.05, n = 3 (independent experimental approaches).

Free intracellular ATP, as the universal energy carrier in the cells, also serves as an indicator of cellular stress. In order to further investigate possible cellular stress due to surface landscapes, the ATP level in cells on the structured cer.face 14 in comparison to the plane Col control was measured with a fluorometric ATP assay kit. MG-63 cells on cer.face 14 indicated no significant changes in their amount of ATP compared with the Col control, the “gold standard” in biocompatibility studies (~1.3-fold) (Figure 7). The cell growth on mild nano-micro curved cer.face 14 profiles was not an energy-consuming process.

## 4. Discussion

Zirconia (ZrO_2_) is the most preferred bioengineering ceramic used in dentistry. The microstructural and physical properties of zirconia ceramics are affected by changes in chemical composition and sintering parameters but also by the grain size and varying quantities of yttria [33]. In other studies, with Y-TZP ceramics from Vita, the energy dispersive spectrometer (EDX) analysis revealed an elemental composition predominantly of Zr (76.4–86.3 wt%), O (9.8–14.6 wt%), Y (3.5–8.1 wt%), and Al (0.2–0.7 wt%) [33]. This is in the range of our qualitative EDX analyses of cer.face^®^14 ceramic of the same company. The addition of oxides such as yttria delays the deleterious phase transformation by stabilizing the tetragonal structure, thus inhibiting its transformation to monoclinic zirconia [34] (p. 146).

A systematic meta-analysis compared titanium with zirconia dental implants with different surface topographies [35], with a focus on bone to implant contact (BIC). No significant difference in the BIC was observed between titanium and the machined zirconia implants. However, a significantly better BIC was observed for acid-etched zirconia implants than for those of titanium. The reason might be in the different surface topography as seen in our Figure 3. The resulting structure after acid etching could depend on the different bulk materials—Ti or zirconia. Finally, the authors suggested that acid-etched zirconia implants may serve as a possible substitute for successful osseointegration [35].

In a previous study, we proved that osteoblasts adhere, grow, and are viable on the cer.face 14 surface [11]. Spreading seems to be slightly impaired due to the nano-micro profile of the ceramic. As we could clearly show here, the cells anchor themselves to the surface and even go into the depth of the cavities, so that the cell area appears somewhat smaller than on smooth surfaces. Furthermore, it could be shown that the osteogenic differentiation (mRNA) was increased after 3 days [11].

For bone remodelling, oxidative stress plays a role. The reactive oxygen species (ROS) is a family of reactive molecules and free radicals generated from oxygen. They are produced during normal aerobic metabolism, notably in the mitochondrion from an incomplete reduction of oxygen by electron transfer [36]. ROS are mainly represented in cells by the superoxide radical anion (O_2_^−^), hydrogen peroxide (H_2_O_2_), and the hydroxyl radical (OH^.^). Depending on their concentration, ROS can either have beneficial or deleterious effects on tissues [37]. To see if our MG-63 cells are generally able to increase their ROS level, we stressed the cells artificially with H_2_O_2_ as positive control. H_2_O_2_ can diffuse through cell membranes, and the fluorescence intensity of DCF, oxidized in the presence of ROS, was strongly increased. This made it obvious that cells growing on cer.face 14 are stress-free.

In addition, the determination of cellular adenosine triphosphate (ATP) of human MG-63 osteoblasts on cer.face 14, compared with collagen-coated glass after 24 h, was an additional indicator for optimal cell growth. Oxidative phosphorylation, the process of energy conversion to ATP, involves the transport of electrons in the mitochondria, which is responsible for most of the ROS production [37].

In earlier studies with corundum-blasted Ti surfaces or Ti pillar structures, we observed an attempted phagocytosis of the sharp-edged elevations, which unnecessarily costs the cells a great deal of energy, as seen by a reduced ATP level and increased ROS production [23,24]. Subsequently, the MG-63 cells were no longer able to function as osteoblasts, i.e., the extracellular matrix protein synthesis of collagen I, bone sialo protein 2, and fibronectin was inhibited [22,25]. The group of Wauquier et al. [37] thoroughly explained the correlation of oxidative stress and bone remodeling. As a result of oxidative stress, the balance of the dynamic bone remodeling is disturbed, and bone formation by osteoblasts is reduced. The underlying mechanism is that osteoblasts under stress are reduced in their capability to produce osteoprotegerin. This protein is an osteoclastogenesis inhibitory factor. The receptor activator of the NF-kB ligand (RANKL) is also expressed by osteoblasts and binds to the pre-osteoclasts. Subsequently, these cells differentiate into multinucleated osteoclasts, which are responsible for normal bone resorption. However, without the decoy receptor osteoprotegerin due to increased ROS, bone loss is dramatically enhanced upon oxidative stress. Our study was able to prove that the cer.face 14 surface did not increase the ROS level in osteoblasts.

## 5. Conclusions

We conclude from our experiments in vitro that cell growth is stress-free for human MG-63 osteoblasts on the acid-etched, yttria-stabilized zirconia surface cer.face 14 due to its mild nano-micro curved topography. In addition, cells are well anchored and integrated into the cer.face 14 structure. An important criterion for the success of dental implants is to find a material that is attractive for osteoblasts and supports the mechanical anchoring of the cells with nano-micro structured surface.

## References

[1] Ritzert B. Zahnimplantate Zunehmend Erste Wahl. https://idw-online.de/de/news707107.

[2] Zhang D., Wong C.S., Wen C., Li Y. (2017). Cellular responses of osteoblast-like cells to 17 elemental metals. J. Biomed. Mater. Res. Part A.

[3] Fage S.W., Muris J., Jakobsen S.S., Thyssen J.P. (2016). Titanium: A review on exposure, release, penetration, allergy, epidemiology, and clinical reactivity. Contact Dermat..

[4] Del Amo F.S.-L., Garaicoa-Pazmiño C., Fretwurst T., Castilho R.M., Squarize C.H. (2018). Dental implants-associated release of titanium particles: A systematic review. Clin. Oral Implant. Res..

[5] Nedjat A. Rem Tene, Verba Sequentur. https://www.dimagazin-aktuell.de/marktplatz/kollegentipps/story/rem-tene-verba-sequentur_7845.html.

[6] Sadowsky S.J. (2020). Has zirconia made a material difference in implant prosthodontics? A review. Dent. Mater..

[7] Thoma D.S., Ioannidis A., Cathomen E., Hämmerle C.H.F., Husler J., Jung R.E. (2016). Discoloration of the Peri-implant Mucosa Caused by Zirconia and Titanium Implants. Int. J. Periodontics Restor. Dent..

[8] Cosgarea R., Gasparik C., Dudea D., Culic B., Dannewitz B., Sculean A. (2014). Peri-implant soft tissue colour around titanium and zirconia abutments: A prospective randomized controlled clinical study. Clin. Oral Implant. Res..

[9] Pieralli S., Kohal R., Jung R., Vach K., Spies B. (2016). Clinical Outcomes of Zirconia Dental Implants: A Systematic Review. J. Dent. Res..

[10] Ritzert B. Materialien Auf Dem Prüfstand: Keramikimplantate. https://www.dginet.de/web/dgi/presse-aktuelles.

[11] Rohr N., Bergemann C., Nebe J.B., Fischer J. (2020). Crystal structure of zirconia affects osteoblast behavior. Dent. Mater..

[12] Nebe B., Heimann R. (2020). In Vitro Studies and Cell Adhesion to Biomaterials. Materials for Medical Applications.

[13] Gruening M., Neuber S., Nestler P., Lehnfeld J., Dubs M., Fricke K., Schnabelrauch M., Helm C.A., Müller R., Staehlke S. (2020). Enhancement of Intracellular Calcium Ion Mobilization by Moderately but Not Highly Positive Material Surface Charges. Front. Bioeng. Biotechnol..

[14] Kirchhof K., Hristova K., Krasteva N., Altankov G., Groth T. (2008). Multilayer coatings on biomaterials for control of MG-63 osteoblast adhesion and growth. J. Mater. Sci. Mater. Med..

[15] Siow K.S., Britcher L., Kumar S., Griesser H.J. (2006). Plasma Methods for the Generation of Chemically Reactive Surfaces for Biomolecule Immobilization and Cell Colonization—A Review. Plasma Process. Polym..

[16] Rebl H., Finke B., Lange R., Weltmann K.-D., Nebe J.B. (2012). Impact of plasma chemistry versus titanium surface topography on osteoblast orientation. Acta Biomater..

[17] Zinger O., Zhao G., Schwartz Z., Simpson J., Wieland M., Landolt D., Boyan B.D. (2005). Differential regulation of osteoblasts by substrate microstructural features. Biomaterials.

[18] Pieuchot L., Marteau J., Guignandon A., Dos Santos T., Brigaud I., Chauvy P.-F., Cloatre T., Ponche A., Petithory T., Rougerie P. (2018). Curvotaxis directs cell migration through cell-scale curvature landscapes. Nat. Commun..

[19] Mörke C., Rebl H., Finke B., Dubs M., Nestler P., Airoudj A., Roucoules V., Schnabelrauch M., Körtge A., Anselme K. (2017). Abrogated Cell Contact Guidance on Amino-Functionalized Microgrooves. ACS Appl. Mater. Interfaces.

[20] Fujita S., Ohshima M., Iwata H. (2009). Time-lapse observation of cell alignment on nanogrooved patterns. J. R. Soc. Interface.

[21] Löffler R., Fleischer M., Kern D.P., Matschegewski C., Stählke S., Nebe B., Lange R. (2012). Pyramid array substrates for biomedical studies. J. Vac. Sci. Technol. B.

[22] Matschegewski C., Staehlke S., Loeffler R., Lange R., Chai F., Kern D.P., Beck U., Nebe J.B. (2010). Cell architecture–cell function dependencies on titanium arrays with regular geometry. Biomaterials.

[23] Staehlke S., Haack F., Waldner A.-C., Koczan D., Moerke C., Mueller P., Uhrmacher A.M., Nebe J.B. (2020). ROS Dependent Wnt/β-Catenin Pathway and Its Regulation on Defined Micro-Pillars—A Combined In Vitro and In Silico Study. Cells.

[24] Moerke C., Mueller P., Nebe B. (2016). Attempted caveolae-mediated phagocytosis of surface-fixed micro-pillars by human osteoblasts. Biomaterials.

[25] Staehlke S., Koertge A., Nebe B. (2015). Intracellular calcium dynamics dependent on defined microtopographical features of titanium. Biomaterials.

[26] Ravindran A.J., Karthigeyan S., Bhat R.T.R., Nageshwarao M.N., Murugesan S.V., Angamuthu V. (2019). Surface modification techniques for zirconia-based bioceramics: A review. J. Pharm. Bioallied Sci..

[27] Bergemann C., Duske K., Nebe J.B., Schöne A., Bulnheim U., Seitz H., Fischer J. (2015). Microstructured zirconia surfaces modulate osteogenic marker genes in human primary osteoblasts. J. Mater. Sci. Mater. Med..

[28] Bailey S., Karsenty G., Gundberg C., Vashishth D. (2017). Osteocalcin and osteopontin influence bone morphology and mechanical properties. Ann. N. Y. Acad. Sci..

[29] Staehlke S., Rebl H., Nebe B. (2019). Phenotypic stability of the human MG-63 osteoblastic cell line at different passages. Cell Biol. Int..

[30] Czekanska E.M., Stoddart M.J., Ralphs J.R., Richards R.G., Hayes J.S. (2013). A phenotypic comparison of osteoblast cell lines versus human primary osteoblasts for biomaterials testing. J. Biomed. Mater. Res. Part A.

[31] Meyer M. (2019). Processing of collagen based biomaterials and the resulting materials properties. Biomed. Eng..

[32] Duske K., Jablonowski L., Koban I., Matthes R., Holtfreter B., Sckell A., Nebe J.B., Von Woedtke T., Weltmann K.-D., Kocher T. (2015). Cold atmospheric plasma in combination with mechanical treatment improves osteoblast growth on biofilm covered titanium discs. Biomaterials.

[33] Sen N., Isler S. (2020). Microstructural, physical, and optical characterization of high-translucency zirconia ceramics. J. Prosthet. Dent..

[34] Heimann R., Heimann R. (2020). Types and Properties of Biomaterials. Materials for Medical Applications.

[35] Hafezeqoran A., Koodaryan R. (2017). Effect of Zirconia Dental Implant Surfaces on Bone Integration: A Systematic Review and Meta-Analysis. BioMed Res. Int..

[36] Lee J.B., Shin Y.M., Kim W.S., Kim S.Y., Sung H.J., Noh I. (2018). ROS-Responsive Biomaterial Design for Medical Applications. Biomimetic Medical Materials. Advances in Experimental Medicine and Biology.

[37] Wauquier F., Leotoing L., Coxam V., Guicheux J., Wittrant Y. (2009). Oxidative stress in bone remodelling and disease. Trends Mol. Med..

